# “Hitting rock bottom” and recovery‐oriented change in long‐standing anorexia nervosa: A case report

**DOI:** 10.1002/pcn5.70384

**Published:** 2026-07-23

**Authors:** Ayumu Kocha, Ryo Watanabe, Atsuo Nakagawa, Hiroki Kocha

**Affiliations:** ^1^ Department of Neuropsychiatry St. Marianna University School of Medicine Kawasaki Japan; ^2^ Department of Psychiatry Sakuragaoka Municipal Hospital Tokyo Japan

**Keywords:** aspiration, case report, hitting rock bottom, long‐standing anorexia nervosa, motivation for recovery, recovery‐oriented cognitive therapy

## Abstract

**Background:**

Long‐standing anorexia nervosa is often associated with severe medical and psychosocial deterioration and difficulty sustaining engagement in treatment. Although “hitting rock bottom” experiences have been described as turning points in recovery from addiction, their clinical significance in long‐standing anorexia nervosa remains poorly understood.

**Case presentation:**

We report the case of a woman in her late thirties with long‐standing anorexia nervosa, binge‐eating/purging type, who had a history of multiple hospitalizations without sustained nutritional restoration. Following COVID‐19 infection and profound malnutrition, she underwent a life‐threatening medical deterioration. This was followed by genuine regret over the life she had lost, a renewed desire to live, and the emergence of a personally meaningful aspiration: to regain her health and be able to play with her child. During hospitalization, supportive care and collaborative exploration of personally meaningful values helped stabilize this aspiration, leading to sustained engagement in treatment and measurable physical improvement from a body mass index of 10.9 at psychiatric admission to 12.9 at discharge.

**Conclusions:**

This case illustrates how, even in long‐standing anorexia nervosa, a “hitting rock bottom” experience was followed by recovery‐oriented change, accompanied by genuine regret, renewed aspiration, and the emergence of a meaningful image of improved health. The process may be understood from the perspective of the aspiration framework described in recovery‐oriented cognitive therapy, as involving not only renewed motivation, but also the restoration of a psychological horizon that allows a healthier future self to become imaginable.

## BACKGROUND

Patients with eating disorders frequently present with profound disturbances in body image and self‐perception. In long‐standing anorexia nervosa, chronic illness progression may lead to marked medical and psychosocial deterioration, diminished insight, and pronounced cognitive rigidity.^1^, ^2^ Conventional psychotherapies may have limited effectiveness when intrinsic motivation for recovery is lacking.

In addiction research and recovery‐oriented change narratives, “hitting rock bottom” has been described not merely as the occurrence of a life‐threatening event, but as a subjective psychological turning point in which individuals recognize that their illness has resulted in intolerable losses and that continuing in the same manner is no longer possible.^3^, ^4^ Although this experience may be precipitated by a medical or psychosocial crisis, it is conceptually distinct from the crisis itself.^3^ Related literature on motivational change and recovery‐oriented change has likewise emphasized turning points characterized by recognition of loss, self‐reflection, and increasing readiness for change.^3^, ^4^

However, although “hitting rock bottom” has been discussed as a catalyst for recovery‐oriented change in addiction, its role in long‐standing anorexia nervosa remains poorly understood. In particular, little is known about how these deeply affecting experiences may translate into renewed motivation, value‐based aspiration, and behavioral change in patients with longstanding anorexia nervosa.

Here, we report the case of a woman with long‐standing anorexia nervosa whose increased engagement in treatment followed a profound “hitting rock bottom” experience during hospitalization. The present case suggests the therapeutic significance of such a psychological turning point, particularly when it is accompanied by the emergence of value‐based aspirations. This process may be understood in light of the aspiration framework described in recovery‐oriented cognitive therapy (CT‐R), which emphasizes future‐oriented identity and personally meaningful goals. To our knowledge, few case reports in long‐standing anorexia nervosa have discussed “hitting rock bottom” experiences in relation to the aspiration construct described in CT‐R.

## CASE PRESENTATION

### Patient information

Written informed consent for publication of this case report, including clinical details and anonymized quotations, was obtained from the patient.

A woman in her late thirties was admitted to the university hospital under medical protection due to profound weight loss, dehydration, and global physical deterioration. Her height was 167 cm and weight 30.4 kg (body mass index [BMI] 10.9).

### Medical and psychiatric history

She had no prior significant medical conditions. Her elder sister was diagnosed with panic disorder, but no family history of eating disorders was reported.

### Developmental and social history

Born in Tokyo as the second of three siblings, she achieved normal developmental milestones. After graduating from high school, she entered university but dropped out in her second year due to difficulty adapting to school life. She later worked in customer service. At age 26, she married and subsequently had one daughter, living with her husband and child.

### Premorbid personality

Her premorbid personality was characterized as polite, conscientious, and highly responsive to meet others’ expectations—traits that may have contributed to vulnerability in self‐evaluation and risk for eating pathology.

### Onset and course

Her eating difficulties began in junior high school, when she was teased for being “fat.” She began restricting food intake and became increasingly absent from school. In high school, she joined a dance club and further intensified her dieting behaviors, later developing binge eating and self‐induced vomiting. Through her twenties, her weight fluctuated around 45 kg. After childbirth in her late twenties, self‐induced vomiting worsened, and her weight again began to decline, ultimately leading to her first involuntary hospitalization for malnutrition at age 30.

Over the following years, she underwent multiple hospitalizations under medical protection but frequently requested early discharge, with her husband generally supporting her decisions. Concerns regarding childcare and separation from her family contributed to these requests. Although she did not completely refuse treatment, the duration and continuity of nutritional rehabilitation were repeatedly limited. Despite repeated warnings regarding the medical risks associated with her condition, she was unable to sustain treatment engagement. Her highest lifetime weight was 65 kg during the first year of junior high school, whereas her lowest weight was in the 26‐kg range immediately prior to the present admission. During previous hospitalizations, she was repeatedly discharged at a body weight close to that at admission, with her weight remaining in the 28‐kg range at the time of her most recent prior discharge. Thus, nutritional restoration comparable to that achieved during the present admission had not been obtained during earlier hospitalizations.

From illness onset to the present admission, the patient had lived with anorexia nervosa for approximately 20 years. Functional impairment became increasingly evident after childbirth. As her physical condition progressively deteriorated, with worsening unsteadiness and muscle weakness, routine household activities such as laundry and housework required substantial effort. She also became unable to play actively with her child, and her range of activity gradually narrowed.

By autumn of the year preceding admission, she had become bedridden and contracted COVID‐19, which further worsened her nutritional status. At that time, COVID‐19 was still widely perceived as a serious and potentially life‐threatening illness. In the context of profound physical deterioration, she was confronted with a condition carrying a risk of sudden clinical worsening.

Unable to eat or walk, she was admitted to the university hospital's intensive care unit (ICU) in a life‐threatening condition requiring pleural drainage and parenteral nutrition. At ICU admission, she weighed 25.9 kg, corresponding to a BMI of 9.2. She presented with dyspnea secondary to pleural effusion and decreased oxygenation, and her condition was considered critical, with a risk of sudden deterioration. After 6 weeks of medical stabilization, she was transferred to the psychiatric unit for psychiatric treatment.

### Mental status and diagnosis

On psychiatric evaluation, she was alert and cooperative but spoke softly, expressing persistent fear of weight gain and ambivalence toward recovery: “I must stay thin, but I know this is killing me.” Although she acknowledged the severity of her condition, she remained hesitant to engage fully in treatment. She met DSM‐5‐TR criteria for anorexia nervosa, binge–purge subtype.

### Clinical course and interventions

Nutritional rehabilitation was initiated at 1550 kcal/day and gradually increased to 2500 kcal/day under multidisciplinary supervision. She initially exhibited marked anxiety about food intake but gradually became more compliant. Her legal status was changed to voluntary admission on day 34. Complications of heart failure and superior mesenteric artery syndrome required temporary caloric restriction but stabilized with medical management.

During this period, she began to engage in emotionally meaningful self‐reflection, particularly regarding how her illness and physical frailty had progressively constrained her relationship with her daughter. Even when they went out together, she was unable to be physically active or play outdoors with her daughter. Although she had strongly wished to do so, this inability became a significant source of emotional distress and inner conflict.

Tearfully, she stated, “I couldn't play with my daughter. I just watched from the car. I don't want to waste my life anymore.” Thereafter, she began articulating a renewed aspiration “to regain health” so she could fulfill her maternal role. This was accompanied by reduced expressed resistance to treatment and greater engagement with nutritional rehabilitation. Her value‐based motivation to live and regain health persisted throughout the remainder of hospitalization.

She was discharged on day 68 weighing 36 kg, corresponding to a BMI of 12.9. No psychotropic medications were prescribed during this period, and improvement was achieved primarily through supportive and nutritional intervention. During the available follow‐up period of several months after discharge, she remained medically stable, managing daily household activities and caring for her child. She reported occasional anxiety related to eating but no purging behaviors or readmission during this period. A timeline summarizing weight trajectory, admission milestones, and the emergence of aspiration during hospitalization is presented in Table 1.

**Table 1:** Timeline of clinical course and psychological change.

Date/Period	Medical Course	Psychological Change
Age 13–15	Onset of dieting and vomiting after being teased for weight	Development of fear of obesity and distorted body image
Age 30–36	Repeated hospitalizations and early discharges	Denial of illness and ambivalence toward treatment
Nov, Year X‐1	COVID‐19 infection and severe weight loss	Physical collapse and recognition of a life‐threatening state
Jan, Year X	Admission under medical protection	Recognition of life‐threatening crisis
Feb, Year X	Transition to voluntary admission	Emergence of regret as a mother and aspiration “to regain health”
Mar, Year X	Discharge at 36 kg (body mass index 12.9)	Sustained motivation for recovery and stable daily functioning
Several months post‐discharge	Medically stable, managing daily household activities and caring for her child	Recovery‐oriented motivation maintained; occasional eating‐related anxiety without purging

*Note*: Timeline of clinical course and psychological change from adolescence to several months post‐discharge.

## DISCUSSION

### The present hospitalization as a “hitting rock bottom” experience

In long‐standing anorexia nervosa, patients frequently encounter recurrent medical crises; however, only a minority convert such crises into meaningful psychological turning points toward recovery‐oriented change.^5^, ^6^ In the present case, a transformative shift emerged under paradoxical conditions—during a period of extreme medical instability in which active psychotherapy was considered inappropriate. Confronted with the possibility of death after COVID‐19 infection, the patient, perhaps for the first time, fully apprehended the severity and consequences of her illness.

The patient appeared to recognize the possibility that she might die, and this realization may have contributed to subsequent changes in treatment engagement. Although many patients with long‐standing anorexia nervosa endure comparable medical dangers, most do not initiate such change, suggesting that motivational transformation requires more than awareness of medical risk alone.^5^, ^6^, ^7^ In this patient, the “hitting rock‐bottom” experience did not simply intensify fear; it appears to have disrupted a long‐standing narrowing of her mental and emotional life, making room for new values and a newly imaginable future.

### The role of genuine regret and aspiration

The pivotal shift in this case was characterized by the simultaneous presence of two contrasting but complementary emotions: genuine regret and aspiration. The predominant affect was a profound sense of genuine regret over her inability to care for her young daughter—a loss that deeply challenged her identity as a mother. This regret was directed not merely toward her physically life‐threatening condition, but toward the way she had lived while prioritizing her fear of weight gain above all else. Importantly, this sorrow did not culminate in hopelessness. Rather, it gave rise to a realistic and future‐oriented desire to live, regain physical health, and reconnect meaningfully with her child.

Aspiration, in turn, provided a new organizing principle for action. It was grounded in personally meaningful values and involved a future‐oriented identity and purpose. Although CT‐R was not applied in this case, the process can be understood from the perspective of its aspiration framework.^7^ The interaction of genuine regret and aspiration appears to have loosened pathological rigidity and fostered a renewed sense of direction. Notably, this motivational reorganization occurred despite the absence of formal psychotherapy, suggesting that in long‐standing anorexia nervosa, profound emotional experiences may occasionally support recovery‐oriented change when they reactivate deeply held personal values and future‐oriented goals.

### Why transformation is rare

From the perspective of the aspiration framework described in recovery‐oriented cognitive therapy, the emergence of aspiration may be understood as the reactivation of personally meaningful values that can support behavioral and emotional change.^7^ The Transtheoretical Model of Change also helps to situate this shift as movement from contemplation toward preparation and action. However, these frameworks alone do not fully explain why only a small proportion of patients with long‐standing anorexia nervosa undergo such a transition even when exposed to similarly severe medical crises.^5^, ^6^

For many patients—particularly those with early‐onset and long‐standing illness—the protracted course of anorexia nervosa progressively erodes opportunities to imagine or pursue a coherent life project. Over time, their identity becomes narrowly defined by the role of being “an eating‐disorder patient,” and the world of possible futures gradually recedes. Thus, what is lost is not merely the capacity to envision recovery, but the chance to do so within a life increasingly organized around the illness. When the horizon of possible selves collapses in this way, neither external motivation nor realistic confrontation with medical risk is sufficient to instigate meaningful change.

The present case illustrates that transformation becomes possible only when an emotionally meaningful image of a healthier future self re‐emerges—an image that restores the capacity to direct one's aspirations toward life itself.^7^ Such re‐expansion of the psychological horizon may be important for recovery‐oriented change in long‐standing anorexia nervosa, helping to explain why genuine turning points remain exceptional despite the ubiquity of medical crises in this population.

### Clinical implications

Although no active intervention was undertaken in the form of a planned or highly directive psychotherapeutic approach, the present case suggests that clinicians may need to recognize and support emerging motivation when it arises spontaneously in patients with long‐standing anorexia nervosa. Rather than prematurely imposing change, clinicians may benefit from adopting a supportive and attentive stance that helps the patients articulate a newly emerging wish “to regain health” and connect it with personally meaningful values. Such an approach may facilitate the restoration of self‐worth and help transform a transient emotional shift into sustained engagement in treatment.

However, even when confrontation with mortality gives rise to a psychological turning point, relapse remains common in long‐standing anorexia nervosa. Accordingly, the emergence of a wish “to regain health” should not be regarded as sufficient in itself. What may be crucial is whether this emerging wish can be elaborated into a personally meaningful and future‐oriented image of the healthier future self.

Continued follow‐up is needed to monitor not only weight and medical stability, but also eating‐disorder‐related anxiety, fear of weight gain, purging behaviors, and potential disengagement from treatment. Ongoing support from both clinicians and family members may play an important role in helping patients sustain this emerging motivation for recovery.

### Limitations and future directions

This report describes a single case, and causality cannot be inferred. Psychometric measures were not administered. In addition, nutritional rehabilitation, medical stabilization, and improvement in the physiological consequences of profound malnutrition may themselves have contributed to changes in cognition, affect, and treatment engagement. Therefore, the observed changes cannot be attributed solely to the patient's reported “hitting rock bottom” experience. The post‐discharge follow‐up period was limited and detailed longitudinal weight data were unavailable; thus, this report describes early recovery‐oriented change rather than sustained recovery. Finally, although the patient had a long illness duration, severe malnutrition, marked functional impairment, and repeated medically necessary hospitalizations, the available retrospective information did not permit a complete assessment of whether she had received an adequate course of specialized outpatient eating‐disorder treatment. We therefore use the term long‐standing anorexia nervosa rather than applying severe and enduring anorexia nervosa as a definitive classification of treatment resistance. Nevertheless, the qualitative trajectory described here offers insight into possible recovery mechanisms in long‐standing anorexia nervosa. Future longitudinal and mixed‐methods studies may help clarify whether similar processes can be therapeutically facilitated within structured treatment programs. The proposed conceptual model requires replication across cases and prospective evaluation.

## CONCLUSIONS

This case illustrates how, even in long‐standing anorexia nervosa, a rock‐bottom experience involving confrontation with severe illness may be associated with recovery‐oriented change when followed by genuine regret, renewed aspiration, and the emergence of a meaningful image of improved health. Table 2 summarizes the proposed psychological process from emotional constriction to recovery‐oriented change. These findings suggest that improvement in long‐standing anorexia nervosa may depend, at least in part, on the re‐emergence of personally meaningful values and a future‐oriented image of improved health.

**Table 2:** Conceptual model of recovery‐oriented change in long‐standing anorexia nervosa.

Stage	Description	Psychological process
1. Chronic fixation	Long‐term dominance of fear of obesity and restrictive eating patterns	Marked narrowing of her psychological world and persistent ambivalence toward recovery
2. Crisis/“hitting rockbottom” experience	Physical collapse and a rock‐bottom experience that brought her face to face with death	Recognition of the seriousness of her condition, with fear and emotional shock
3. Emergent self‐reflection	Recognition of what had been lost, especially in relation to her role as a mother	Emergence of genuine regret and renewed self‐observation
4. Activation of aspiration	Desire to live, recover, and reconnect with her child	Emergence of a valued future self and a personally meaningful wish “to regain health”
5. Sustained engagement	Behavioral engagement with treatment and stabilization of motivation	Integration of this wish into everyday functioning and recovery‐oriented behavior

*Note*: A conceptual model illustrating the stepwise transition from chronic fixation to recovery‐oriented engagement in long‐standing anorexia nervosa, emphasizing the roles of the “hitting rock bottom” experience, genuine regret, self‐reflection, and aspiration.

## AUTHOR CONTRIBUTIONS

Ayumu Kocha and Ryo Watanabe drafted the manuscript and treated the patient. Atsuo Nakagawa and Hiroki Kocha provided supervision and conceptual input. All authors read and approved the final manuscript.

## CONFLICT OF INTEREST STATEMENT

The authors declare no conflicts of interest.

## ETHICS APPROVAL STATEMENT

Not applicable. According to our institutional policy, single‐patient case reports do not require IRB approval.

## PATIENT CONSENT STATEMENT

Written informed consent for publication (clinical details and anonymized quotations) was obtained from the patient. Documentation is retained by the corresponding author and will be provided on request.

## CLINICAL TRIAL REGISTRATION

N/A.

## Data Availability

Data sharing not applicable to this article as no datasets were generated or analyzed during the current study. De‐identified materials (e.g., timeline and minimal clinical descriptors) are available from the corresponding author on reasonable request, subject to institutional policy and the patient's consent. No publicly shareable dataset was generated for this case report.

## References

[1] Marcolini F , Ravaglia A , Tempia Valenta S , Bosco G , Marconi G , De Ronchi D , et al. Severe enduring anorexia nervosa (SE‐AN) treatment options and their effectiveness: a review of literature. J Eat Disord. 2024;12(1):48. 10.1186/s40337-024-01006-y 38654374 PMC11040982

[2] Wonderlich JA , Dodd DR , Sondag C , Jorgensen M , Blumhardt C , Evanson AN , et al. Clinical and scientific review of severe and enduring anorexia nervosa in intensive care settings: introducing an innovative treatment paradigm. J Eat Disord. 2024;12(1):131. 10.1186/s40337-024-01079-9 39227928 PMC11373466

[3] Kirouac M , Frohe T , Witkiewitz K . Toward the operationalization and examination of “hitting bottom” for problematic alcohol use: a literature review. Alcohol Treat Q. 2015;33(3):312–327.

[4] Chen G . Building recovery capital: the role of “hitting bottom” in desistance and recovery from substance abuse and crime. J Psychoactive Drugs. 2018;50(5):420–429. 30204568 10.1080/02791072.2018.1517909

[5] Eddy KT , Tabri N , Thomas JJ , Murray HB , Keshaviah A , Hastings E , et al. Recovery from anorexia nervosa and bulimia nervosa at 22‐year follow‐up. J Clin Psychiatry. 2017;78(2):184–189. 10.4088/JCP.15m10393 28002660 PMC7883487

[6] Wonderlich SA , Peterson CB , Crosby RD , Engel SG , Mitchell JE , Smyth J , et al. Psychological treatments for people with severe and enduring eating disorders. Front Psychiatry. 2020;11:206. 10.3389/fpsyt.2020.00206 32265758 PMC7106475

[7] Beck AT , Grant PM , Inverso E , Brinen AP , Perivoliotis D . Recovery‐oriented. In: Cognitive therapy for serious mental health conditions. Guilford Press; 2020.

